# Potential crosstalk of oxidative stress and immune response in poultry through phytochemicals — A review

**DOI:** 10.5713/ajas.18.0538

**Published:** 2018-10-26

**Authors:** M. T. Lee, W. C. Lin, T. T. Lee

**Affiliations:** 1 Department of Animal Science, National Chung Hsing University, Taichung 402, Taiwan; 2 The iEGG and Animal Biotechnology Center, National Chung Hsing University, Taichung 402, Taiwan

**Keywords:** Phytochemicals, Oxidative Stress, Immune Response, Poultry

## Abstract

Phytochemicals which exist in various plants and fungi are non-nutritive compounds that exert numerous beneficial bioactive actions for animals. In recent years following the restriction of antibiotics, phytochemicals have been regarded as a primal selection when dealing with the challenges during the producing process in the poultry industry. The selected fast-growing broiler breed was more fragile when confronting the stressors in their growing environments. The disruption of oxidative balance that impairs the production performance in birds may somehow be linked to the immune system since oxidative stress and inflammatory damage are multi-stage processes. This review firstly discusses the individual influence of oxidative stress and inflammation on the poultry industry. Next, studies related to the application of phytochemicals or botanical compounds with the significance of their antioxidant and immunomodulatory abilities are reviewed. Furthermore, we bring up nuclear factor (erythroid-derived 2)-like 2 (Nrf2) and nuclear factor kappa B (NF-κB) for they are respectively the key transcription factors involved in oxidative stress and inflammation for elucidating the underlying signal transduction pathways. Finally, by the discussion about several reports using phytochemicals to regulate these transcription factors leading to the improvement of oxidative status, heme oxygenase-1 gene is found crucial for Nrf2-mediated NF-κB inhibition.

## INTRODUCTION

Phytochemicals are non-nutritive yet powerful compounds derived from natural resources like plants and fungi [1–4]. Based on the chemical structure that endows several bioactive actions, phytochemicals are classified into four groups such as polyphenols, terpenoids, alkaloids, and sulfur-containing phytochemicals [5]. Despite being regarded as non-essential for typical body functioning, their health-promoting and disease-relieving effects in mammal studies have made them the subject of promising natural feed additives in poultry industry, especially after the prohibition of using antibiotics by European Union in 2006 [6,7].

Several factors are responsible for compromising productive and reproductive performance, as well as welfare of animals. These include various stressors and inflammatory responses that are recognized as the major contributors and thus have drawn lots of attention in recent years [8,9]. In 2017, Gessner et al [10] reviewed the antioxidative and anti-inflammatory abilities of plant polyphenols and advanced the possible linkage between oxidative stress and inflammation in farm animals. Besides, our recent two review articles, which discussed the potential of antioxidant and anti-inflammation effects of phytochemicals in animals respectively, also suggest that there exists an inseparable relationship between oxidative stress and inflammation [2,11].

Interestingly, the potential crosstalk between oxidation and inflammation has been discussed in numerous mammal studies, particularly for fathoming the pathological processes of specific disease, or evaluating the effectiveness of some drugs or materials in curing some ailments [12–15]. Despite being as a novel research topic in the field of farm animals, these mammal studies provide a detailed platform for referral.

In view of the fact that stress is a multi-step process that can be stimulated and activated by various factors [6], and oxidative stress and inflammatory damage are multi-stage processes [16], and more importantly, the protective effects exerted by any positive nutritional alteration are likely to be the sum of several distinct mechanisms; aiming at components of signal transduction pathways turns out to be a promising way to study this process. Transcription factors are protein complexes that regulate the transcription of genetic information from DNA to mRNA by binding to specific DNA sequences and acting downstream of signaling cascades in response to biological and environmental stimuli [17]. Therefore, transcription factors are effective in the initiation, stimulation or termination of the genetic transcriptional process [18].

The above context demonstrates that many components of the cell signaling network would converge on the transcription factors. By tracing those upstream and downstream molecules, we could simply clarify the pathways involved in specific conditions. Based on the urgency to tackle the severity of oxidative stress in animal production, focusing on oxidative-related genes are of importance. Transcriptional regulation of oxidative-related genes is predominantly mediated by two redox-sensitive transcription factors – nuclear factor (erythroid-derived 2)-like 2 (Nrf2) and nuclear factor kappa B (NF-κB). In our recent research articles [19], we found that medical fungus—*Antrodia cinnamomea* (*A. cinnamomea*) fermented products containing phytochemicals like triterpenoid and phenolics could effectively improve lipopolysaccharide (LPS) and 2,2′-azobis(2-amidinopropane) dihydrochloride caused diminished chicken cell viability *in vitro*, Nrf2 and its downstream antioxidant genes were up-regulated by *A. cinnamomea*, and NF-κB ruled inflammatory genes rather showed opposite expression. Activation of Nrf2 and suppression of NF-κB were further supported in protein level. These suggested that the immunomodulatory and the antioxidant mechanisms were closely intertwined, and agents like phytochemicals with such modulatory effects worth be used to explore the possible crosstalk in oxidative stress and immunomodulation in poultry.

Considering the above stances, this review article discusses the existing and promising antioxidant and immunomodulatory effects exerted by phytochemicals, and further demonstrates phytochemicals induced molecular changes in Nrf2 and NF-κB pathways provide a promising aspect and method to explore such interaction. Developing effective and pleiotropic ways to solve or relieve these complex situations becomes a novel challenge in the modern husbandry industry.

## CHALLENGES CONFRONTING POULTRY INDUSTRY: OXIDATIVE STRESS AND INFLAMMATION

The concept of stress was firstly introduced by Selye [20], defined as “a nonspecific response of the body to any demand made upon it”. Nevertheless, its definition has been constantly revised and developed by other scientists. In 2011, Koolhaas et al [21] furthered the idea about stress, suggesting that events perceived as negative, and where the response exceeds the existing adaptive capacity in the organism, should be regarded as stress. Stress is an inevitable experience for domestic birds in present poultry husbandry system, commercial poultry production is facing various stress factors causing compromised productive and reproductive performance of growing chickens, parent birds, as well as layer hens [22]. Despite of the fact that the stress response stands as an inbuilt security system, alerting animals on possible dangers; successive acute or chronic stress can, in the long run, be detrimental.

Oxidative stress, defined as an imbalanced condition between oxidants formation and elimination by cellular antioxidant system, has been regarded as one of the most pesky issues in the modern poultry industry [6,9,10], owing to the fact that the source of these oxidants varies, from normal metabolism like byproducts of mitochondrial respiration and other physiological metabolism in tissues [9,16], to an externally triggered form like immune response-activated superoxide radicals from polymorphonuclear leukocytes and other phagocytes, and radiation exposure-produced hydroxyl radicals [9,23]. While developing, oxidative stress tends to cause damage to lipids, DNA, proteins and other cell constituents, resulting in severe disruption of cell integrity or even tissue damage [2,9,10].

For the contributing conditions, heat stress induced oxidative stress is the major concern, for not only the inborn limitation of heat dissipation capacity and firm feather, but also the distribution of poultry industries into hot climate areas coupled with the aggravation of global warming [24–26]. Moreover, this situation may be worsening and accompanied by the quality of feed spoiling due to storage under increasing hot and humid environments [27,28]. Being one of the main agricultural industries, poultry production requires fast growing broiler chickens to fulfill the meat market. However, apart from the advantages like short harvesting period and large breast muscles, such genetic selected breeds are extremely sensitive to oxidative stress [29,30].

Inflammation is a fundamental and localized physiological process that is often caused and is accompanied by the imbalance of the oxidative status in the animal’s body. It is a protective reaction to injurious stimuli in the form of infection, trauma, pathogenic invasions, and physical, chemical, or thermal stress. Inflammatory response is a series of dynamic coordinated reactions consisting of specific vascular, humoral and cellular events that are characterized by the movement of fluids, plasma and inflammatory leukocytes (including neutrophils, basophiles and macrophages) to the site of inflammation [11,31].

In the animal body, inflammation process is like a double-edged sword. Potential pathological situations are inevitable in modern farm animal industry where once exposed to external stimuli a moderate inflammatory response is necessary to protect animals from instant threat [8]. However, inflammation process requires a large amount of energy to support such an acute immune response. Lee et al [32] used LPS to challenge chickens (1 mg/kg body weight [BW]), finding that birds with such a challenge would have significant BW loss. Moreover, Jiang et al [33] found that LPS injection would reduce up to 22% BW gain, 41% of the loss derived from factors attending immune response, and the remaining 59% due to decreased feed intake. Other infections like *Eimeria tenella* showed similar adverse effects. Gavage administration of *Eimeria tenella* (40,000 sporulated oocysts at 21 day) would cause nearly 10% weight loss [34]. Accordingly, nutrients will be diverted from growth and productive purposes to support prompt immunological reactions. At the same time, an acute phase response occurs and is responsible for appetite suppression [8,35]. These will reduce profitability and product quality, even endanger animal health [36]. Moreover, genetic selection for pursuing optimal growth performance of commercial broiler lines was reported to have led to a weaker immune potential, as building robust immune response will partition energy for growth [35]. With crowded and intensive rearing patterns in modern animal system, inflammation is intensified and occurs frequently, especially ever since the ban of some antibiotics [10,36,37]. The above stance suggests how important to strike a balance between performance and immune strengthening. Inflammation is a fundamental and localized physiological process and the body’s protective reaction to injurious stimuli in the form of infection, trauma, pathogenic invasions, and physical, chemical, or thermal stress.

With the limited defense abilities of adaptive systems [9], domestic avian husbandry requires prompt and effective ways to establish robust defense systems in order to promote productive and growth performance in the industry.

### Amelioration effects of phytochemicals on oxidative status and immunosuppression in poultry

Table 1 displayed the modulation effects of various phytochemicals on oxidative status and immunosuppression in poultry which will be discussed sequentially in detail in the following text.

#### Grape pomace

Grape pomace is mainly the residual product of the wine industry [38]. With the increasing attention on the bioactive components of industrial byproducts, phenolics in grape pomace is widely studied in research and applied in practice, especially for its strong free radical scavenging action and oxidative retardation diminishing effect. Furthermore, a promising immunomodulating potential derived from the function of phenolics in grape pomace is also highlighted especially in aiding chickens to fight against Newcastle disease virus (NDV) [39,40]. Previous literature showed that supplementation of 60 g/kg pomace in control diet reduced (p<0.05) malondialdehyde (MDA) concentration in the muscle tissue of broilers [41]. Such reduction of oxidative products in chicken meat could ameliorate the possible rancidity of chicken meat. Iqbal et al [40] investigated the potential of grape polyphenols as the alternative to vitamin E for its antioxidant and immune response modifying activities. Results suggested that total phenolic levels in the breast and leg muscles of broiler chickens linearly increased from 50 to 75 ppm, accompanied by decreasing thiobarbituric acid amount. Moreover, with either low or high levels of grape polyphenols (25 and 75 ppm), antibody titers against NDV increased in comparison with the corresponding control group. At the same time, histopathological examination showed no adverse changes in livers and kidneys of chickens receiving 25 ppm grape polyphenols in diet, suggesting that grape polyphenols could stimulate production of antibodies against bacteria without doing harm to the host immune organs. Similarly, Ebrahimzadeh et al [39] found that 7.5% addition of grape pomace improve serum antioxidant indexes such as superoxide dismutase (SOD, 161.5 vs 110.9 U/mL), glutathione peroxidase (GPx, 179.01 vs 133.8 U/mL) activity and MDA concentration (4.83 vs 5.97 nmol/mL) when compared to a vitamin E supplementation group. In the same study, administrating NDV to evaluate the corresponding antibody titer in 42-day old broiler chickens showed that 7.5% grape pomace group had a lower level than the others. However, the secondary titer against sheep red blood cells (SRBC) increased in 10% grape pomace group, outweighing the corresponding control group.

#### Turmeric (Curcuma longa)

Curcumin is the major bioactive polyphenolic compound of Turmeric rhizomes that regulates the antioxidant and immune status through its strong antioxidant activity. Some studies took it as a solution to deal with the negative effects caused by the intake of aflatoxin contaminated feed in poultry industry. Yarru et al [42] investigated the potential of turmeric to ameliorate the negative effects of chickens fed with aflatoxin. Results showed that 0.5% turmeric powder (74 mg/kg of curcumin in feed) supplementation was able to alleviate hepatic SOD and glutathione S-transferase (GST) genes suppressed by aflatoxin challenge. The increased proinflammatory interleukin (IL)-6 gene expression caused by aflatoxin supplementation had also been down-regulated due to its anti-inflammatory activity. Gowda et al [43] also reported that Aflatoxin B1 (AFB1) challenged chicken supplemented with turmeric powder containing 74, 222, and 444 mg/kg of total curcuminoids had increased serum antioxidant functions compared to the non-supplemented challenged group. In the same study, 444 mg/kg total curcuminoids supplemented group without challenge had increased (p<0.05) serum total antioxidant level compared to the non-challenged control group (44.2 vs 22.1 mM/mL). Gowda et al [44] also reported that 0.5% turmeric powder supplementation in broiler feed elevated the serum albumin concentration (from 0.5 to 0.9 g/dL). Rajput et al [45] found that dietary supplementation of 200 mg/kg curcumin would increase the Newcastle disease and avian influenza titers more than the control group, and such effects were even greater than that achieved with lutein, another phytochemical-rich material in this experiment. Moreover, since lymphocyte proliferation is another indicator for examining cellular immunity in chickens, enhanced B and T lymphocyte proliferation in spleen occurred in curcumin containing group which suggested that curcumin has immunomodulatory effects on the immune organs.

#### Pleurotus mushrooms

Mushrooms have been used as food for centuries, for their subtle flavor and high nutritional value [4,46]. Their therapeutic and protective benefits against diseases are currently a hot-topic, especially for their antioxidant, immunomodulating, and even antitumor effects [46–48]. Similar to those well-known beneficial plants, mushrooms also contain great number of bioactive phytochemicals like phenolics, polysaccharides, and organic acids that make them as descent candidate of natural pharmaceutical agents [47,49,50].

*Pleurotus eryngii* (*P. eryngii*, King Oyster mushroom), standing as the third most cultivated mushroom world-wide, owns a promising antioxidant potentiality, attributed to its secondary metabolites like phenolic compounds and polysaccharides [51–53]. Our previous study suggested that the stalk residue of *P. eryngii* (PESR) possesses total phenolics (about 5 mg/g dry weight [DW]) and crude triterpenoid (1.84 mg/g DW); and as compared to a control group, 0.5%, 1.0%, and 2.0% PESR addition increased antioxidant enzymes level including SOD and catalase (CAT), along with diminished MDA amount in serum or breast of broiler chickens [51]. On the other hand, *Pleurotus ostreatus* (*P. ostreatus*), commonly known as Oyster mushroom, is also an edible mushroom that possess biological properties similar to *P. eryngii*. Vargas-Sánchez et al [54] showed that total phenolic content and flavonoid in the *P. ostreatus* were 30.0 g/kg and 25.0 g/kg respectively, and for the polysaccharide content was 340.0 g/kg in *P. ostreatus*. In addition, when *P. ostreatus* was added to a quail diet, its total phenolic content was 55.2% higher than the control diet, to the results were 32.6% and 43.0% 2,2-Diphenyl-1-picrylhydrazyl and 2,2′-azino-bis(3-ethylbenzothiazoline-6-sulphonic acid) free radical scavenging effects as compared with the basal diet. It’s noticeable that without compromising growth performance, meat quality parameters in terms of L* (9.0%) and thiobarbituric acid reactive substances value (33.5%) were reduced (p<0.05) in quails that received *P. ostreatus* containing diet in comparison with the control group; all of these were reported to have great possibility to be associated with the phenolics and polysaccharides in the *P. ostreatus*. Apart from antioxidant benefits, mushrooms also have immunomodulatory and anti-inflammatory activities [55,56]. Mushroom polysaccharides were extensively studied for their immunomodulatory effects in both mammal and avian studies [57,58]. Polysaccharides derived from *P. ostreatus* and *Pleurotus sajor-caju* increased antibody titers like total immunoglobulin (Ig), IgM, and IgG at the 7th and 14th day after primary and secondary injections of SRBC. Additionally, these polysaccharide extracts were able to reduce oocyst count resulted from mixed species of genus *Eimeria* infection [59]. On the other hand, our recent review article demonstrated the important role of the gastrointestinal tract in defending against latent immunosuppressive stressors in animal body [11], suggesting intestinal parameters are potential evaluating indexes of immune health status in animals. Polysaccharides in oyster mushroom were reported to contribute to increase in villus height and crypt depth of jejunum when 1% and 2% oyster mushroom were included in diet. Moreover, the lowest level of oyster mushroom inclusion (1% in diet) could cause marginal improvement of antibody titers against influenza disease virus and SRBC [60].

#### Ganoderma lucidum

*Ganoderma lucidum* (*G. lucidum*) is an oriental fungus that has been used in Traditional Chinese Medicine for more than 2000 years. In the Chinese history, it’s called “lingzhi” that symbolizes its essence of immortality [61]. Recent scientific research has supported the ancient claims of its spiritual health benefits as its pharmacological effects include antioxidant, anti-inflammation, antibacterial, and immunomodulatory actions which are mainly derived from their active components like polysaccharides and triterpenes [61–63]. Since Aflatoxin is considered one of the most rampant mycotoxins in the poultry production, several literatures have used AFB1, a pro-oxidant agent of Aflatoxins, to generate oxidative stress and immune suppression to investigate the ameliorating effects exerted by dietary supplementation of *G. lucidum*. For example, with dietary addition of AFB1, chickens in this group showed pronounced increased level of H_2_O_2_ and MDA in liver along with suppressed serum immunoglobulins, yet such negative effects were improved while supplementing *G. lucidum* in the diet. Moreover, antioxidant defense systems were evaluated through hepatic CAT, glutathione reductase (GR), GPx, and glutathione activities. On the other hand, serum IgG and IgA were elevated compared to the non-supplemented challenged group. Results showed that *G. lucidum* supplementation in AFB1-contaminated diet could effectively enhance levels of AFB1-induced suppression of these indexes [64]. AL-Zuhariy et al [65] showed that after 45 days of AFB1 administration, birds fed 0.2% *G. lucidum* supplemented diet had the lowest hepatic and splenic H_2_O_2_ and MDA concentration in comparison with those received that AFB1-added control diet. Moreover, GR and GPx antioxidant indices in either liver or spleen were improved (p<0.05) by *G. lucidum* addition as well. The improvement in antioxidant capacities and activities observed in this study was attributed to the radical-removing ability of polysaccharides in *G. lucidum*. Moreover, they applied Newcastle disease (ND) vaccination to evaluate the immune-boosting efficacy of *G. lucidum* against ND under the toxic effects induced by AF1 in chickens of different ages. In the successive harvest day (the third, fourth and fifth weeks), birds with *G. lucidum* plus AFB1 in diet showed the highest antibody titer; and with the challenge of virulent NDV isolate (100 ELD_50_ 10^5^), *G. lucidum* could increase antibody titer of immune antibodies. These studies suggested that polysaccharides in *G. lucidum* holds potentiality to mitigate immunosuppression and elevate the antioxidant status to alleviate the negative effects cause by the consumption of AFB1 in poultry industry.

#### Forsythia suspense

*Forsythia suspense* (FS) is a medicinal plant that is distributed in China, Japan and Korea. Recently, its antioxidant effects have been widely studied. Wang et al [66] reported that broilers in finisher phase fed with FS extracts (100 mg/kg) had greater serum total antioxidant capacity and reduced MDA concentration compared with the control group when challenged with high ambient temperature. Furthermore, average gain and feed conversion ration were also improved in the birds supplemented with FS extracts. Corticosterone (CS) has been commonly used as a stimulant to trigger acute stress in broiler. Broilers supplemented with FS extracts (100 mg/kg) have been reported to alter the average daily gain (ADG) and feed conversion ratio impairment caused by CS. In addition, MDA accumulation in serum has also been reduced followed by the by enhanced serum SOD and total antioxidant capacity in FS extracts supplemented group [67]. Pan et al [68] had also discovered the improved drip loss, MDA and carbonyl contents in breast muscle of CS challenged broilers supplemented with FS extracts (100 mg/kg). CS-induced reduction of total-antioxidant capacity, GPx, and SOD in breast muscle were also alleviated by the supplementation of FS extracts. Traditionally people applied the dried fruit extracts of *Forsythia suspensa* to treat inflammation-related diseases [69]. In poultry industry, FS extract also showed potential to improve broiler performance through its immunomodulating effects. Zang et al [70] supplemented broilers with FS extracts in a 42-d feeding trial under high stocking density (46 kg of BW/m^2^). Results showed that birds fed 100 mg/kg of FS extracts had greater bursa weight and bursa weight/BW ratio, along with improved final BW, ADG, and average daily feed intake compared with control group. Furthermore, FS extracts supplementations elevated the decreased antibody titers to NDV causing by CS challenge and increased relative weights of thymus. Forsythiaside has been identified as the main bioactive compound in fruits of FS. Cheng et al [71] indicated that forsythiaside supplementation (30 or 60 mg/kg) can attenuate LPS-induced inflammation by inhibiting tumor necrosis factor-α, IL-6, IL-1β, and cyclooxygenase-2 (COX-2) production via suppressing NF-κB in the bursa of Fabricius of chickens.

## POTENTIAL TRANSCRIPTION FACTORS LINKING OXIDATIVE STRESS AND INFLAMMATION IN POULTRY

### Nuclear factor (erythroid-derived 2)-like 2

Nrf2 serves as the chief transcription factor orchestrating antioxidant response in terms of binding to antioxidant response element (ARE) located in the promoter region, transcribing genes encoding phase II detoxifying antioxidant enzymes and several detoxifying proteins, including GST, heme oxygenase-1 (HO-1), GPx, and glutamate cysteine ligase (GCL), NAD(P)H:quinine oxidoreductase, peroxiredoxin 1 [72,73].

**Figure 1:**
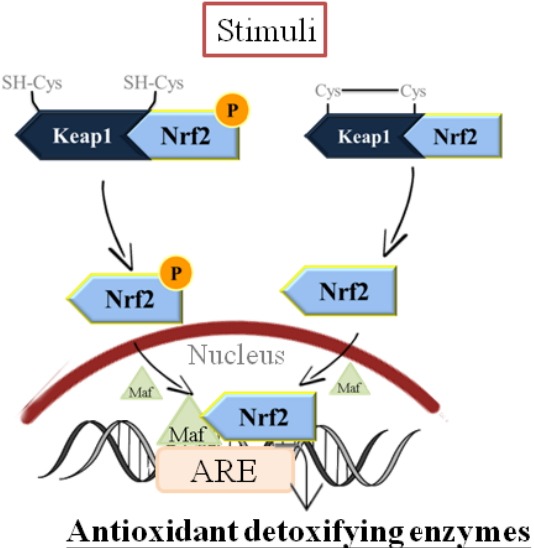
Nuclear factor (erythroid-derived 2)-like 2 (Nrf2) serves as the chief transcription factor orchestrating antioxidant response in terms of binding to antioxidant response element (ARE) located in the promoter region, transcribing genes encoding phase II detoxifying antioxidant enzymes and several detoxifying proteins.

As illustrated in Figure 1, under basal conditions, Nrf2 is sequestered in the cytoplasm by its repressor protein, Kelch-like ECH-associated protein 1 (Keap1). In response to oxidative stress, there is a disruption of the interaction between Nrf2 and Keap1, and then Nrf2 will translocate from the cytoplasm to the nucleus, and sequentially binds to ARE, leading to a cytoprotective response which is characterized by up-regulation of a group of antioxidant enzymes and decreased sensitivity to oxidative damage [74,75].

Antioxidants derived from Nrf2 are defined as indirect antioxidants in that their physiological effects last longer than those being exerted by direct antioxidants, suggesting that with relatively low-dosage, they can exert sufficient efficacy. Besides, a high-dosage direct antioxidant like vitamin E had been found to stimulate pro-oxidant actions, yet these are unlikely to happen in indirect antioxidants [76].

Therefore, applying materials possessing the ability to activate Nrf2 signaling would be promising diet supplements in poultry husbandry regarding their long-term antioxidant effects and effectiveness.

### Nuclear factor kappa B

**Figure 2:**
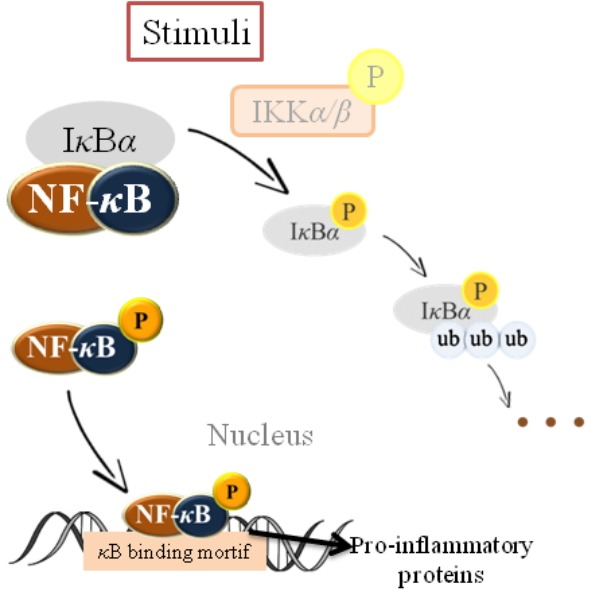
Inflammatory responses to a wide variety of stimuli mainly attribute to up-regulation of the proinflammatory transcription factor - nuclear factor kappa B (NF-κB). Since it is a kind of redox-sensitive transcription factor, NF-κB responses to a number of stimuli including reactive oxygen species under normal circumstances, NF-κB is sequestered in the cytoplasm by binding to the inhibitory protein called inhibitor of kappa B (IκB). Besides, the IκB kinase (IKK) complex is the signal integration hub for NF-κB activation, its main role is integrating signals from all NF-κB activating stimuli to catalyze the phosphorylation of various IκB and NF-κB proteins. After activation by stress, diet alteration, free radicals, inflammatory stimuli, cytokines and the presence of carcinogens, NF-κB translocates to the nucleus, and then induces the expression of different inflammatory cytokines and many other genes related to cellular transformation, invasion, metastasis and inflammation.

Inflammatory responses to a wide variety of stimuli mainly attribute to up-regulation of the proinflammatory transcription factor (NF-κB) [77]. Since it is a kind of redox-sensitive transcription factor, NF-κB responses to several stimuli including reactive oxygen species (ROS) [16,78]. As shown in Figure 2, under normal circumstances, NF-κB is sequestered in the cytoplasm by binding to the inhibitory protein called inhibitor of kappa B (IκB). Besides, the IκB kinase (IKK) complex is the signal integration hub for NF-κB activation, its mainly role is integrating signals from all NF-κB activating stimuli to catalyze the phosphorylation of various IκB and NF-κB proteins, as well as of other substrates. After activated by stress, diet alteration, free radicals, inflammatory stimuli, cytokines and the presence of carcinogens, NF-κB translocates to the nucleus, and then induces the expression of different inflammatory cytokines and chemokines, enzymes such as cyclooxygenase (COX2) and nitric oxide synthase, and many other genes related to cellular transformation, invasion, metastasis and inflammation [79,80].

In the field of animal production, a recent study demonstrated that increased expression of NF-κB was found in heat-stressed quail [81,82], which partially proved that stress-like hyperthermal treatment may induce NF-κB activation [67,78]. Report showed that increasing dietary Epigallocatechin gallate (EGCG) extracted from green tea reduced 42% NF-κB expression in the hepatic cells of quail treated by heat stress. Moreover, NF-κB p65 subunit level increased in the nuclear fraction of the liver from heat stressed quails, and tomato powder supplementation attenuated this response in a dose-dependent manner [73]. Hence, phytochemicals possessing potentiality to suppress NF-κB signaling pathway may be a good candidates to exercise anti-stress effects.

## INSEPARABLE ISSUES: OXIDATIVE STRESS AND INFLAMMATION

**Figure 3:**
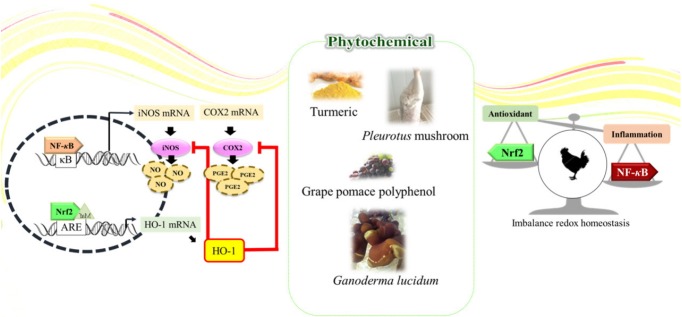
Potential crosstalk between oxidative stress and inflammation through phytochemicals in poultry.

Numerous literatures demonstrate and support an interdependent relationship between inflammation and oxidative stress [12,14]. For instance, the activated phagocytic cells like neutrophils and macrophages during inflammation produce large amounts of ROS, reactive nitrogen species, and reactive carbonyl species to eradicate invading agents. Exaggerated generation of reactive species may burst out under pathological inflammatory conditions, and some of those reactive species diffuse out of the phagocytic cells and thus they can induce localized oxidative stress and tissue injury [83]. Apart from the direct production of reactive species by the specific phagocytic cells, the nonphagocytic cells can also produce reactive species in response to proinflammatory cytokines [13,15]. Several studies have applied antioxidants to activate immune system and enhance antioxidant capacity to protect chickens from AFB1 toxicity [65,84]. Moreover, in the case of the heat stress situation in poultry production, high temperature induced oxidative stress is associated with not only compromised antioxidant status and ability, but also suppressed immune functions in terms of secretion of inflammatory markers and decreased level of related vitamins and minerals in animal body [6,81,82]. ROS generated by heat stress would disrupt membrane electron transport, and further modulates the activation of Nrf2 and NF-κB, driving the change of molecules involving in oxidation and immune actions (Figure 3) [6,85,86]. The above studies drop a hint that the inflammatory process can induce oxidative stress, and the oxidative stress can also stimulate inflammation by activating multiple pathways. Viewed from the therapeutic angle, it is noteworthy that there is a positive correlation between anti-inflammatory activities of phytochemicals with their ability to induce antioxidant gene expression.

### Potential crosstalk between oxidative stress and inflammation through phytochemicals in poultry

Sahin et al [87] reported that supplementation of tomato powder could inhibit NF-κB activity in heat-stressed quail, accompanied by increased nuclear Nrf2 translocation. This might be attributed to the phytochemical, lycopene, in the tomato that is capable of activating Nrf2 [88]; the antioxidant enzymes like SOD, CAT, and GPx were also augmented, showing that dietary inclusion of tomato powder would boost Nrf2 antioxidant activity and thus reduce inflammatory-related NF-κB activation. Similarly, EGCG, the most abundant catechin in various type of tea, when directly supplemented in quail diet, demonstrated that this phytochemical was also a potential Nrf2 activator and NF-κB suppressor. Furthermore, indexes like antioxidant enzymes (CAT, SOD, and GPx) were linearly increased in quail fed a EGCG including diet. Feed intake was also elevated from 29.6 to 30.9 g/d, and Nrf2 and NF-κB expression increased and decrease in linear way as well. This study further showed that oxidative biomarkers had a strong correlation with hepatic Nrf2 and NF-κB expression, suggesting EGCG could alleviate oxidative stress induced by high ambient temperature in quail. Decreased SOD, CAT, and GPx activities along with suppressed Nrf2 and augmented NF-κB expression in quails exposed to heat stress were improved by dietary supplementation with resveratrol and curcumin [81, 82], two phytochemicals well proved to have cytoprotective effects in medical research [89–91]. It’s interesting to find that both studies found that heat shock protein, one of the members in vitagene family, expression was increased in the liver of quails suffering a high temperature environment. The vitagene family has been regarded as a promising resolving strategy to fight against detrimental effects caused by oxidative imbalance [16,86] In addition to heat shock proteins, thioredoxins (Trx)/thioredoxin reductase (TrxR) system, SOD, and sirtuins are members that preserve homeostasis in cell, to adapt cells in various stress conditions [92]. Among those candidates for combating NF-κB in Nrf2-regulated pathway, HO-1 is at the core of Nrf2-mediated NF-κB inhibition, and HO-1 is one of the heat shock family members [93]. Sahin et al [82] showed that as Nrf2 activated by curcumin, the downstream *HO-1* gene expression was induced and thus be proposed as contributor to NF-κB suppression. Interestingly, our recent published research supplementing powder of *A. cinnamomea*, one of the renowned medical mushrooms, in broiler chicken diet showed that apart from significant serum antioxidant potentiality improved, Nrf2 dominated genes like HO-1, glutamate cysteine ligase catalytic were up-regulated in 35-day old chickens, accompanied by suppressed expression of NF-κB and its downstream inflammatory genes. These were evidenced in protein level of the two master transcription factors as well [19]. The most important point lies on the fact that; those results of ameliorated physiological indexes were reflected on the better performance indices as well. However, it is noteworthy that we could scarcely find literatures to support or corroborate this possible relationship in broiler chicken. Since broiler meat is one of the major animal protein sources undergoing a rapidly increasing demand [88]. Moreover, attention has been put on improving animal welfare [94], showing not only that the intensity of broiler rearing condition should be addressed, but also that prevention of excessive oxidative stress in the broiler husbandry is imperative.

## CONCLUSION

Phytochemicals derived from plants and mushrooms are known for their bioactive effects, including antioxidation and anti-inflammation, which have been extensively studied for a long time. It’s noteworthy that correlation between ability of phytochemicals to induce antioxidant actions and suppress inflammatory damage has been gaining attention and thereby being widely applied in various pathological conditions in human research, providing a strong support for farm animal studies aimed at uncovering their crosstalk. Oxidative stress resulting from diversified factors internally or externally can be severe and inflammation can be regarded as a byproduct. To dissect such an intertwined correlation, transcription factors includes Nrf2 and NF-κB dominated antioxidative and inflammatory pathways are useful targets for studying their interfaces. Currently, more and more literature in the poultry field has demonstrated possible interactions in these two pathways. Therefore, to prevent an increasing level of oxidative stress and inflammation, understanding molecular changes induced by phytochemicals could help formulate mechanistic pathways and develop more precise ways to solve severe oxidative stress and inflammation in the present poultry industry.

## Figures and Tables

**Table 1:** Amelioration effects of phytochemicals on oxidative status and immune responses in poultry

Phytogenic materials	Supplemented form	Effective dosages	Challenge	Effects on immune responses	Effects on oxidative status	References
Grape pomace	Grape pomace	60 g/kg diet	Unchallenged		Lowered MDA in muscle tissue	[41]
	Grape polyphenols	25–75 ppm in the diet	Unchallenged	Increased antibody titers against NDV	Increased polyphenol levels and decreased TBA amount in breast and leg muscles	[40]
	Grape pomace	7.5% in the diet	Unchallenged	Lowered antibody titer against NDV, higher secondary titer against SRBC	Higher serum SOD and GPx; lowered serum MDA	[39]
Turmeric (*Curcuma longa*)	Powder	0.5% in the diet	Aflatoxin B1	Lowered IL-6 mRNA expression, higher serum albumin concentration	Alleviate hepatic SOD, GST mRNA expression	[42], [44]
	Powder	74, 222, and 444 mg/kg curcuminoids in the diet	Aflatoxin B1		Increased serum antioxidant functions	[43]
	Powder	444 mg/kg curcuminoids in the diet	Unchallenged		Higher serum T-AOC	[43]
	powder	200 mg/kg curcumin in the diet	Unchallenged	Increased antibody titers against NDV, enhanced B and T lymphocyte proliferation in spleen		[45]
*Pleurotus* mushrooms	Stalk residue powder	0.5%, 1.0%, and 2.0% in the diet	Unchallenged		Higher SOD, CAT, lowered MDA in serum & muscle	[51]
	Dried *Pleurotus ostreatus*	10g/kg and 20 g/kg in the diet	Unchallenged		Lowered TBARS value in breast muscle at d15 of storage test	[54]
	Mushroom derived polysaccharides	25 mg/kg BW	Eimeria *infection*	Reduced *Eimeria* oocyst count		[59]
			Unchallenged	Increased total Ig, IgM, and IgG titers against SRBC		
	Oyster mushroom	1% in the diet	Unchallenged	increased antibody titers against influenza disease virus and SRBC		[60]
*Ganoderma lucidum*	Sporoderm-broken spores	200 mg/kg in the diet	Aflatoxin B1	Increased IgG and IgA	Lowered H2O2 and MDA, higher CAT, GR, GPx and GSH in liver	[64]
	Powder	0.2% in the diet	Aflatoxin B1	Increased antibody titers against NDV	Lowered H2O2 and MDA, higher GR and GPx in liver and spleen	[65]
*Forsythia suspense*	Extracts	100 mg/kg in the diet	Heat		Greater serum total antioxidant capacity and reduced MDA concentration	[66]
	Extracts	100 mg/kg in the diet	Corticosterone		Lowered MDA, higher SOD in serum	[67]
	Extracts	100 mg/kg in the diet	Corticosterone		Lowered MDA and carbonyl contents, higher T-AOC, GPx, and SOD in breast muscle	[68]
	Extracts	100 mg/kg in the diet	High stocking density	Greater bursa weight and bursa weight/BW ratio, increased antibody titers against NDV		[70]
	Forsythiaside	30 and 60 mg/kg in the diet		Inhibit TNF-α, IL-6, IL-1β, and COX-2 production via suppressing NF-κB in the bursa of Fabricius of chickens		[71]

BW, body weight; MDA, malondialdehyde; NDV, Newcastle disease virus; TBA, thiobarbituric acid; SRBC, sheep red blood cell; SOD, superoxide dismutase; GPx, glutathione peroxidase; T-AOC, total-antioxidant capacity; IL, interleukin; GST, glutathione S-transferase; Ig, immunoglobulin; CAT, catalase; TBARS, thiobarbituric acid reactive substances; GR, glutathione reductase; GSH, glutathione; TNF, tumor necrosis factor; COX, cyclooxygenase; NF, nuclear factor.

## References

[1] Kamboh AA, Khan MA, Kaka U (2018). Effect of dietary supplementation of phytochemicals on immunity and haematology of growing broiler chickens. Ital J Anim Sci.

[2] Lee MT, Lin WC, Yu B, Lee TT (2017). Antioxidant capacity of phytochemicals and their potential effects on oxidative status in animals — A review. Asian-Australas J Anim Sci.

[3] Unekwu HR, Audu JA, Makun MH, Chidi EE (2014). Phytochemical screening and antioxidant activity of methanolic extract of selected wild edible Nigerian mushrooms. Asian Pac J Trop Dis.

[4] Wandati TW, Kenji GM, Onguso JM (2013). Phytochemicals in edible wild mushrooms from selected areas in Kenya. J Food Res.

[5] Barbieri R, Coppo E, Marchese A (2017). Phytochemicals for human disease: An update on plant-derived compounds antibacterial activity. Microbiol Res.

[6] Sahin K, Orhan C, Smith MO, Sahin N (2013). Molecular targets of dietary phytochemicals for the alleviation of heat stress in poultry. Worlds Poult Sci J.

[7] Valenzuela-Grijalva NV, Pinelli-Saavedra A, Muhlia-Almazan A, Domínguez-Díaz D, González-Ríos H (2017). Dietary inclusion effects of phytochemicals as growth promoters in animal production. J Anim Sci Technol.

[8] Broom LJ, Kogut MH (2018). Inflammation: friend or foe for animal production?. Poult Sci.

[9] Surai PF (2015). Antioxidant systems in poultry biology: superoxide dismutase. J Anim Res Nutr.

[10] Gessner DK, Ringseis R, Eder K (2017). Potential of plant polyphenols to combat oxidative stress and inflammatory processes in farm animals. J Anim Physiol Anim Nutr.

[11] Huang CM, Lee TT (2018). Immunomodulatory effects of phytogenics in chickens and pigs — a review. Asian-Australas J Anim Sci.

[12] Castellani P, Balza E, Rubartelli A (2014). Inflammation, DAMPs, tumor development, and progression: a vicious circle orchestrated by redox signaling. Antioxid Redox Signal.

[13] Li J, Lan T, Zhang C (2015). Reciprocal activation between IL-6/ STAT3 and NOX4/Akt signalings promotes proliferation and survival of non-small cell lung cancer cells. Oncotarget.

[14] Mittal M, Siddiqui MR, Tran K, Reddy SP, Malik AB (2014). Reactive oxygen species in inflammation and tissue injury. Antioxid Redox Signal.

[15] Wu Y, Lu J, Antony S (2013). Activation of TLR4 is required for the synergistic induction of dual oxidase 2 and dual oxidase A2 by IFN-γ and lipopolysaccharide in human pancreatic cancer cell lines. J Immunol.

[16] Calabrese V, Cornelius C, Mancuso C (2008). Cellular stress response: A novel target for chemoprevention and nutritional neuroprotection in aging, neurodegenerative disorders and longevity. Neurochem Res.

[17] Latchman DS (1997). Transcription factors: an overview. Int J Biochem Cell Biol.

[18] Haddad JJ (2002). Science review: Redox and oxygen-sensitive transcription factors in the regulation of oxidant-mediated lung injury: role for nuclear factor-κB. Crit Care.

[19] Lee MT, Lin WC, Wang SY (2018). Evaluation of potential antioxidant and anti-inflammatory effects of *Antrodia cinnamomea* powder and the underlying molecular mechanisms via Nrf2- and NF-κB-dominated pathways in broiler chickens. Poult Sci.

[20] Selye H (1936). A syndrome produced by diverse nocuous agents. Nature.

[21] Koolhaas JM, Bartolomucci A, Buwalda B (2011). Stress revisited: A critical evaluation of the stress concept. Neurosci Biobehav Rev.

[22] Surai PF, Fisinin VI (2016). Vitagenes in poultry production: Part 3. Vitagene concept development. World’s Poult Sci J.

[23] Mruk DD, Silvestrini B, Mo MY, Cheng CY (2002). Antioxidant superoxide dismutase - a review: its function, regulation in the testis, and role in male fertility. Contraception.

[24] Deeb N, Cahaner A (2002). Genotype-by-environment interaction with broiler genotypes differing in growth rate. 3. Growth rate and water consumption of broiler progeny from weight-selected versus nonselected parents under normal and high ambient temperatures. Poult Sci.

[25] Lara LJ, Rostagno MH (2013). Impact of heat stress on poultry production. Animals (Basel).

[26] Yahav S, Straschnow A, Luger D (2004). Ventilation, sensible heat loss, broiler energy, and water balance under harsh environmental conditions. Poult Sci.

[27] Ismail IB, Al-Busadah KA, El-Bahr SM (2013). Oxidative stress biomarkers and biochemical profile in broilers chicken fed zinc bacitracin and ascorbic acid under hot climate. Am J Biochem Mol Biol.

[28] Zhang ZW, Wang QH, Zhang JL (2012). Effects of oxidative stress on immunosuppresion induced by selenium deficiency in chickens. Biol Trace Elem Res.

[29] Nie CX, Zhang WJ, Wang YG (2015). Tissue lipid metabolism and hepatic metabolomic profiling in response to supplementation of fermented cottonseed meal in the diets of broiler chickens. J Zhejiang Univ Sci B.

[30] Sihvo HK, Immonen K, Puolanne E (2014). Myodegeneration with fibrosis and regeneration in the pectoralis major muscle of broilers. Vet Pathol.

[31] Hou DX, Luo D, Tanigawa S (2007). Prodelphinidin B-4 3′-O-gallate, a tea polyphenol, is involved in the inhibition of COX-2 and iNOS via the downregulation of TAK1-NF-κB pathway. Biochem Pharmacol.

[32] Lee Y, Lee S, Gadde UD, Oh S, Lillehoj HS (2017). Relievable effect of dietary *Allium hookeri* on LPS induced intestinal inflammation response in young broiler chickens. J Immunol.

[33] Jiang Z, Schatzmayr G, Mohnl M, Applegate TJ (2010). Net effect of an acute phase response—Partial alleviation with probiotic supplementation. Poult Sci.

[34] Aziza A, Awadin W (2018). Impact of dietary supplementation of whole flaxseed and flaxseed meal to infected broiler chickens with *Eimeria tenella*. Asian J Anim Vet Adv.

[35] Humphrey BD, Klasing KC (2004). Modulation of nutrient metabolism and homeostasis by the immune system. World’s Poult Sci J.

[36] Ahmed SM, Luo L, Namani A, Wang XJ, Tang X (2017). Nrf2 signaling pathway: pivotal roles in inflammation. Biochim Biophys Acta Mol Basis Dis.

[37] Cheng G, Zhao Y, Li Y (2014). Forsythiaside attenuates lipopolysaccharide-induced inflammatory responses in the bursa of Fabricius of chickens by downregulating the NF-κB signaling pathway. Exp Ther Med.

[38] Fontana AR, Antoniolli A, Bottini R (2013). Grape pomace as a sustainable source of bioactive compounds: extraction, characterization, and biotechnological applications of phenolics. J Agric Food Chem.

[39] Ebrahimzadeh SK, Navidshad B, Farhoomandl P, Mirzaei Aghjehgheshlagh F (2018). Effects of grape pomace and vitamin E on performance, antioxidant status, immune response, gut morphology and histopathological responses in broiler chickens. S Afr J Anim Sci.

[40] Iqbal Z, Kamran Z, Sultan JI (2015). Replacement effect of vitamin E with grape polyphenols on antioxidant status, immune, and organs histopathological responses in broilers from 1- to 35-d age. J Appl Poult Res.

[41] Brenes A, Viveros A, Goñi I (2008). Effect of grape pomace concentrate and vitamin E on digestibility of polyphenols and antioxidant activity in chickens. Poult Sci.

[42] Yarru LP, Settivari RS, Gowda NK (2009). Effects of turmeric (*Curcuma longa*) on the expression of hepatic genes associated with biotransformation, antioxidant, and immune systems in broiler chicks fed aflatoxin. Poult Sci.

[43] Gowda NKS, Ledoux DR, Rottinghaus GE, Bermudez AJ, Chen YC (2009). Antioxidant efficacy of curcuminoids from turmeric (*Curcuma longa L*.) powder in broiler chickens fed diets containing aflatoxin B1. Br J Nutr.

[44] Gowda NKS, Ledoux DR, Rottinghaus GE, Bermudez AJ, Chen YC (2008). Efficacy of Turmeric (*Curcuma longa*), containing a known level of curcumin, and a hydrated sodium calcium aluminosilicate to ameliorate the adverse effects of aflatoxin in broiler chicks. Poult Sci.

[45] Rajput N, Naeem M, Ali S (2013). The effect of dietary supplementation with the natural carotenoids curcumin and lutein on broiler pigmentation and immunity. Poult Sci.

[46] Ribeiro B, Valentão P, Baptista P, Seabra RM, Andrade PB (2007). Phenolic compounds, organic acids profiles and antioxidative properties of beefsteak fungus (*Fistulina hepatica*). Food Chem Toxicol.

[47] Kozarski M, Klaus A, Jakovljevic D (2015). Antioxidants of edible mushrooms. Molecules.

[48] Yang JH, Lin HC, Mau JL (2002). Antioxidant properties of several commercial mushrooms. Food Chem.

[49] Valentão P, Lopes G, Valente M (2005). Quantitation of nine organic acids in wild mushrooms. J Agric Food Chem.

[50] Yildiz O, Can Z, Laghari AQ, Sahin H, Malkoc M (2015). Wild edible mushrooms as a natural source of phenolics and antioxidants. J Food Chem.

[51] Lee TT, Ciou JY, Chiang CJ, Chao YP, Yu B (2012). Effect of Pleurotus eryngii stalk residue on the oxidative status and meat quality of broiler chickens. J Agric Food Chem.

[52] Li S, Shah NP (2013). Effects of various heat treatments on phenolic profiles and antioxidant activities of *Pleurotus eryngii* extracts. J Food Sci.

[53] Zhang A, Li X, Xing C, Yang J, Sun P (2014). Antioxidant activity of polysaccharide extracted from *Pleurotus eryngii* using response surface methodology. Int J Biol Macromol.

[54] Vargas-Sáncheza RD, Torrescano-Urrutiab GR, Ibarra-Ariasc FJ (2018). Effect of dietary supplementation with *Pleurotus ostreatus* on growth performance and meat quality of Japanese quail. Livest Sci.

[55] Chen J, Mao D, Yong Y (2012). Hepatoprotective and hypolipidemic effects of water-soluble polysaccharidic extract of *Pleurotus eryngii*. Food Chem.

[56] Jeong YT, Jeong SC, Gu YA, Islam R, Song CH (2010). Antitumor and immunomodulating activities of endo-biopolymers otained from a submerged culture of *Pleurotus eryngii*. Food Sci Biotechnol.

[57] Guo FC, Kwakkel RP, Williams BA (2004). Effects of mushroom and herb polysaccharides on cellular and humoral immune responses of *Eimeria tenella* infected chickens. Poult Sci.

[58] Li X, Jiao LL, Zhang X (2008). Anti-tumor and immunomodulating activities of proteoglycans from mycelium of Phellinus nigricans and culture medium. Int Immunopharmacol.

[59] Ullah MI, Akhtar M, Iqbal Z, Muhammad F (2014). Immunotherapeutic activities of mushroom derived polysaccharides in chicken. Int J Agric Biol.

[60] Fard SH, Toghyani M, Tabeidian SA (2014). Effect of oyster mushroom wastes on performance, immune responses and intestinal morphology of broiler chickens. Int J Recycl Org Waste Agric.

[61] Gill BS, Navgeet, Kumar S (2017). *Ganoderma lucidum* targeting lung cancer signaling: a review. Tumor Biol.

[62] Huang S, Mao J, Ding K (2017). Polysaccharides from *Ganoderma lucidum* promote cognitive function and neutral progenitor proliferation in mouse model of Alzheimer’s disease. Stem Cell Reports.

[63] Zhang B, Yan L, Li Q (2018). Dynamic succession of substrate-associated bacterial composition and function during *Ganoderma lucidum* growth. Peer J.

[64] Liu T, Ma Q, Zhao L (2016). Protective effects of sporoderm-broken spores of *Ganderma lucidum* on growth performance, antioxidant capacity and immune function of broiler chickens exposed to low level of Aflatoxin B_1_. Toxins (Basel).

[65] AL-Zuhariy MTB, Hassan WH (2017). Hepatoprotective and immunostimulatory effect of *Ganoderma*, *Andrographolide* and *Turmeric* against Aflatoxicosis in broiler chickens. Int J Poult Sci.

[66] Wang L, Piao XL, Kim SW (2008). Effects of *Forsythia suspensa* extract on growth performance, nutrient digestibility, and antioxidant activities in broiler chickens under high ambient temperature. Poult Sci.

[67] Zeng ZK, Li QY, Piao XS (2014). Forsythia suspensa extract attenuates corticosterone-induced growth inhibition, oxidative injury, and immune depression in broilers. Poult Sci.

[68] Pan L, Zhao PF, Ma XK (2018). *Forsythia suspensa* extract protects broilers against breast muscle oxidative injury induced by corticosterone mimicked pre-slaughter acute stress. Poult Sci.

[69] Piao XL, Jang MH, Cui J, Piao X (2008). Lignans from the fruits of Forsythia suspensa. Bioorg Med Chem Lett.

[70] Zhang HY, Piao XS, Zhang Q (2013). The effects of Forsythia suspensa extract and berberine on growth performance, immunity, antioxidant activities, and intestinal microbiota in broilers under high stocking density. Poult Sci.

[71] Cheng G, Zhao Y, Li H (2014). Forsythiaside attenuates lipopolysaccharide-induced inflammatory responses in the bursa of Fabricius of chickens by downregulating the NF-κB signaling pathway. Exp Ther Med.

[72] Lee JM, Johnson JA (2004). An important role of Nrf2–ARE pathway in the cellular defense mechanism. J Biochem Mol Biol.

[73] Nguyen T, Nioi P, Pickett CB (2009). The Nrf2-antioxidant response element signaling pathway and its activation by oxidative stress. J Biol Chem.

[74] Cardozo LF, Pedruzzi LM, Stenvinkel P (2013). Nutritional strategies to modulate inflammation and oxidative stress pathways via activation of the master antioxidant switch Nrf2. Biochimie.

[75] Jin W, Wang H, Yan W (2008). Disruption of Nrf2 enhances upregulation of nuclear factor-kappaB activity, proinflammatory cytokines, and intercellular adhesion molecule-1 in the brain after traumatic brain injury. Mediators Inflamm.

[76] Jung KA, Kwak MK (2010). The Nrf2 system as a potential target for the development of indirect antioxidants. Molecules.

[77] Oeckinghaus A, Hayden MS, Ghosh S (2011). Crosstalk in NF-*κ*B signaling pathways. Nat Immunol.

[78] Gilmore TD (2006). Introduction to NF-kappaB: players, pathways, perspectives. Oncogene.

[79] Aggarwal BB, Shishodia S (2006). Molecular targets of dietary agents for prevention and therapy of cancer. Biochem Pharmacol.

[80] Gupta SC, Sundaram C, Reuter S, Aggarwal BB (2010). Inhibiting NF-κB activation by small molecules as a therapeutic strategy. Biochim Biophys Acta.

[81] Sahin K, Orhan C, Akdemir F (2012). Resveratrol protects quail hepatocytes against heat stress: modulation of the Nrf2 transcription factor and heat shock proteins. J Anim Physiol Anim Nutr.

[82] Sahin K, Orhan C, Tuzcu Z, Tuzcu M, Sahin N (2012). Curcumin ameloriates heat stress via inhibition of oxidative stress and modulation of Nrf2/HO-1 pathway in quail. Food Chem Toxicol.

[83] Fialkow L, Wang Y, Downey GP (2007). Reactive oxygen and nitrogen species as signaling molecules regulating neutrophil function. Free Radic Biol Med.

[84] Li Y, Ma QG, Zhao LH (2014). Protective efficacy of α-lipoic acid against aflatoxinB1-induced oxidative damage in the liver. Asian-Australas J Anim Sci.

[85] Ali S, Mann DA (2004). Signal transduction via the NF-kappaB pathway: a targeted treatment modality for infection, inflammation and repair. Cell Biochem Funct.

[86] Sahin N, Tuzcu M, Orhan C (2009). The effects of vitamin C and E supplementation on heat shock protein 70 response of ovary and brain in heat-stressed quail. Br Poult Sci.

[87] Sahin K, Orhan C, Akdemir F (2011). Tomato powder supplementation activates Nrf-2 via ERK/Akt signaling pathway and attenuates heat stress-related responses in quails. Anim Feed Sci Technol.

[88] Ben-Dor A, Steiner M, Gheber L (2005). Carotenoids activate the antioxidant response element transcription system. Mol Cancer Ther.

[89] Farombi EO, Shrotriya YS, Na HK, Kim SH, Surh YJ (2008). Curcumin attenuates dimethylnitrosamine-induced liver injury in rats through Nrf2-mediated induction of heme oxygenase-1. Food Chem Toxicol.

[90] Rubiolo JA, Mithieux G, Vega FV (2008). Resveratrol protects primary rat hepatocytes against oxidative stress damage: activation of the Nrf2 transcription factor and augmented activities of antioxidant enzymes. Eur J Pharmacol.

[91] Sankar P, Telang AG, Manimaran A (2012). Protective effect of curcumin on cypermethrin-induced oxidative stress in Wistar rats. Exp Toxicol Pathol.

[92] Grigorieva MA, Belichko OA, Shabaldin SV, Fisinin VI, Surai PF (2017). Vitagene regulation as a new strategy to fight stresses in poultry production. Agric Biol.

[93] Wardyn JD, Ponsford AH, Sanderson CM (2015). Dissecting molecular cross-talk between Nrf2 and NF-κB response pathways. Biochem Soc Trans.

[94] de Jonge J, van Trijp HC (2013). The impact of broiler production system practices on consumer perceptions of animal welfare. Poult Sci.

